# Temporal bone CT-based anatomical parameters associated with the development of cholesteatoma

**DOI:** 10.1007/s11547-023-01677-8

**Published:** 2023-08-03

**Authors:** Michele Cavaliere, Lorenzo Ugga, Armando Monfregola, Fabrizia Cavaliere, Ferdinando Caranci, Carmela Russo, Francesco Briganti, Andrea Elefante

**Affiliations:** 1 Department of Neurosciences, Reproductive and Odontostomatological Sciences, University of Naples “Federico II”, Naples, Italy; 2 Department of Advanced Biomedical Sciences, University of Naples “Federico II”, Naples, Italy; 3 Department of Precision Medicine, University of Campania “Luigi Vanvitelli”, Piazza Luigi Miraglia, 2, 80138 Naples, Italy; 4 Department of Neurosciences, Unit of Neuroradiology, Santobono-Pausilipon Children’s Hospital, Naples, Italy

**Keywords:** Multidetector computed tomography, Cholesteatoma, Cross-sectional anatomy, Diagnostic imaging

## Abstract

**Background:**

Cholesteatoma is caused by disorders of the middle ear ventilation that trigger a progressive series of events responsible for its formation. The aim of this study was to identify possible radiological CT-derived parameters predisposing to ventilation disorders and cholesteatoma.

**Methods:**

In this retrospective study, patients diagnosed with cholesteatomatous chronic otitis media who underwent temporal bone CT and open tympanoplasty surgery have been included, as well as control patients with clinical examination negative for organic otological pathology who underwent temporal bone CT for other reasons. For each patient, the following parameters have been extracted from CT volumes: degree of mastoid pneumatization, prominence of the cog, patency of the Eustachian tube, antrum width, aditus width, anterior and posterior epitympanic widths, and epitympanic height.

**Results:**

Sixty patients have been included, thirty of whom belonged to the group of patients with cholesteatoma and the remaining part to the group of patients without organic otological pathology. The prevalence of a low degree of mastoid pneumatization was significantly higher among patients with cholesteatoma, as well as for the prevalence of cog prominence (*p* < 0.001). All the continuous variables were found to have statistical significance (*p* < 0.05) in the comparison between groups except for the width of the antrum.

**Conclusion:**

Mastoid pneumatization degree, prominence of the cog and epitympanic measures based on temporal bone CT could be good radiological correlates of the ventilatory capabilities of the epitympanum which, if compromised, can facilitate the development of cholesteatoma.

## Introduction

Cholesteatoma is caused by disorders of the middle ear ventilation that trigger a progressive series of events (formation of retraction pockets, entry of keratinocytes into the tympanic cavity, etc.), which are responsible for its formation [1–4]. This ventilation can be compromised when three conditions occur:

1. Reduced transmucosal diffusion of gas exchange between blood and tympano-mastoid air spaces, as can occur in cases of poor mastoid pneumatization and/or thickening/metaplasia of the lining epithelium [5].
2. Dysfunction and/or non-patency of the Eustachian tube (e.g., obstruction at the nasopharyngeal and/or tympanic level) [6].
3. Obstruction of ventilatory currents between the meso- and epitympanum (narrowing of the tympanic isthmus, presence of tensor folds, etc.), between the anterior and posterior epitympanum (prominence of the cog), and between the posterior epitympanum and the mastoid antrum (narrowing of the aditus) [7, 8].

In light of this evidence, the aim of our study was to identify possible radiological parameters, particularly those derived from CT, capable of predicting the risk of generating ventilation disorders and predisposing the middle ear to the development of cholesteatoma.

## Methods

We conducted a retrospective study including patients admitted to our Institution between 2021 and 2022, belonging to the following two groups. In the first group, patients diagnosed with cholesteatomatous chronic otitis media who underwent open tympanoplasty surgery were selected. All patients underwent high-resolution volumetric computed tomography (CT) [9] and magnetic resonance imaging (MRI) with diffusion-weighted sequences [10–13] and calculation of the ADC parameter [14, 15] during the preoperative workup to confirm the diagnosis of cholesteatoma. Only the affected ear was included in the study. The second group included patients with a negative history and objective examination for organic otological pathology, who underwent temporal bone CT for other reasons (tinnitus, sensorineural hearing loss without middle ear abnormalities, or head trauma with suspected involvement of the temporal bone). Only one ear (by convention the left one) was included in the study. All CT volumetric acquisitions have been obtained using a 64-channel multidetector scanner (tube voltage: 120 kVp, tube current: 250 mA, slice thickness: 0.6 mm, pitch factor: 0.625, rotation time: 0.75 s). A filtered back-projection was employed to reconstruct temporal bone CT axial images and coronal reformats with the bone kernel FC 80 (FOV: 10 cm, matrix: 512 × 512).

The study was approved by the local ethical committee. Written informed consent was preliminarily obtained from all patients included in the study.

**Fig. 1:**
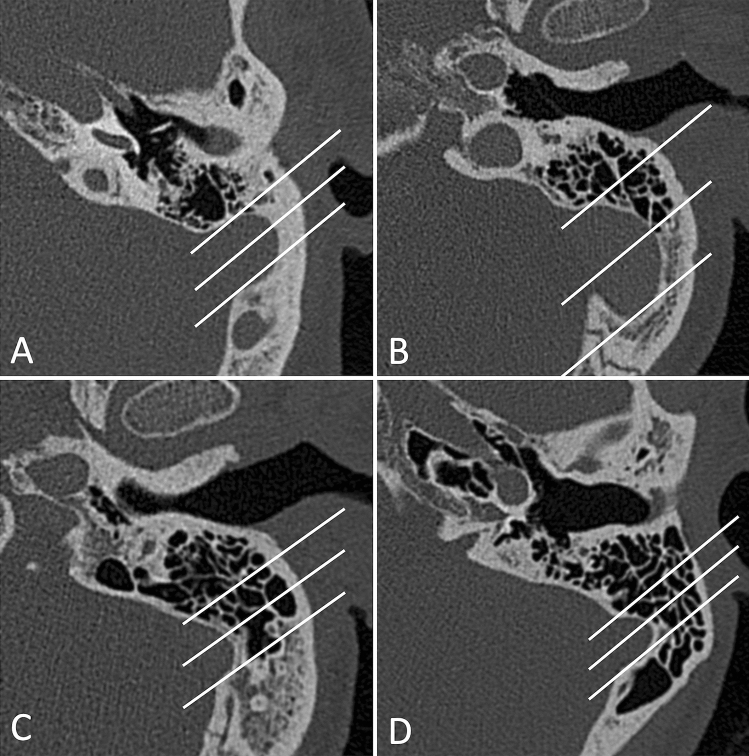
Classification into four grades of mastoid pneumatization **A** hypopneumatization, **B** moderate pneumatization, **C** good pneumatization, **D** hyperpneumatization), according to Han et al. [17]

**Fig. 2:**
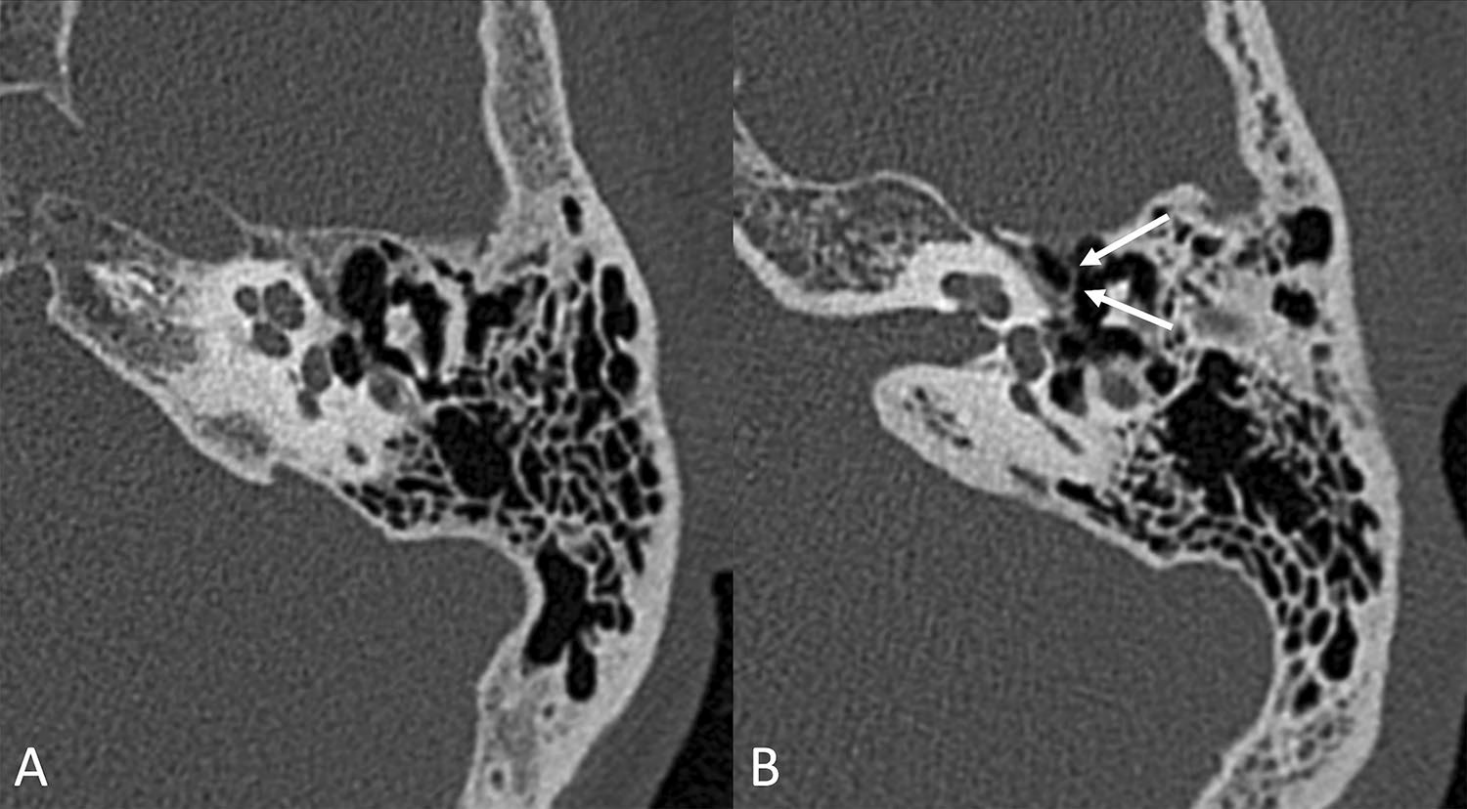
Examples of a non-prominent **A** and a prominent **B** cog at the level of the cochleariform eminence

**Fig. 3:**
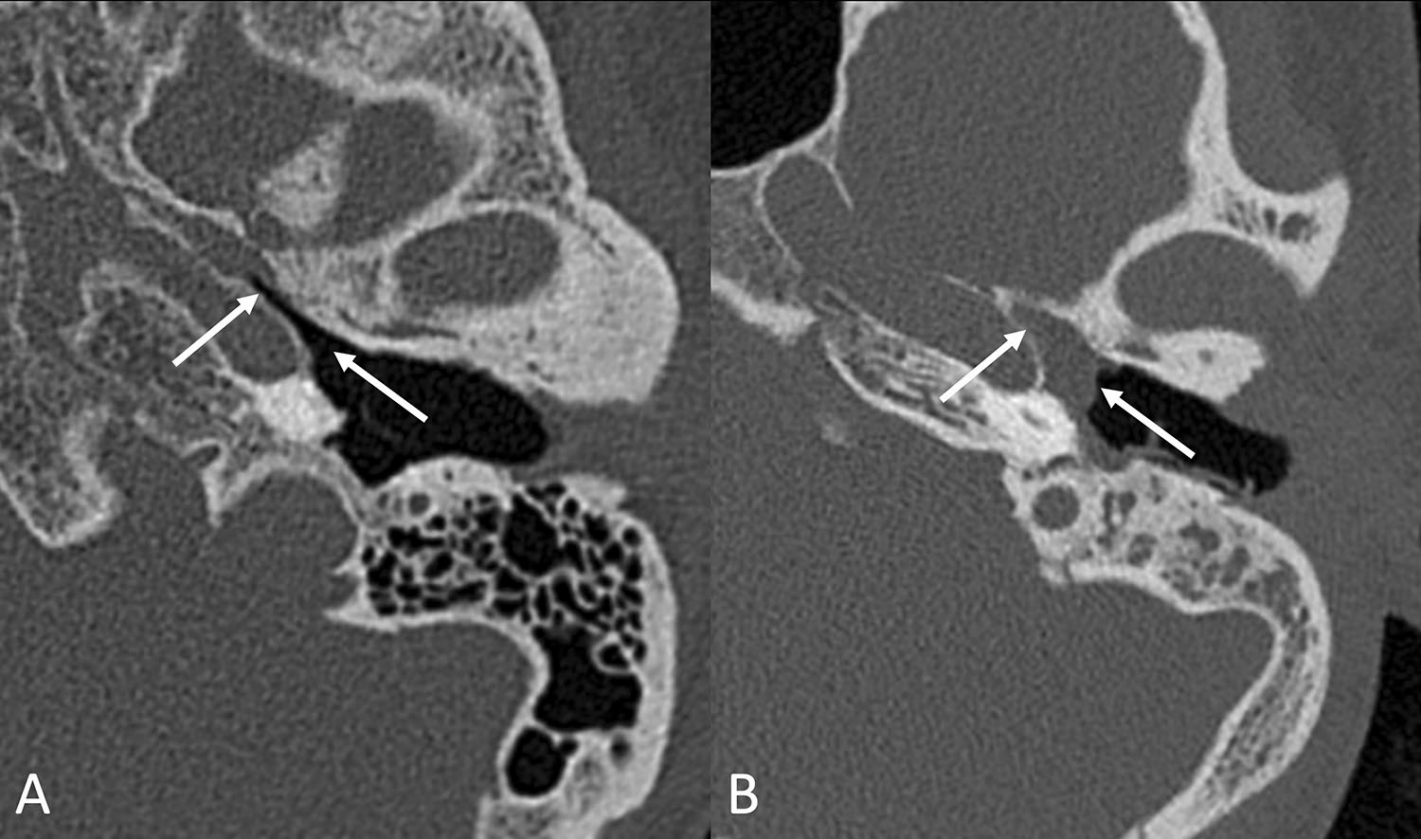
Examples of a pervious **A** and obliterated **B** Eustachian tube at the level of the bony portion

**Fig. 4:**
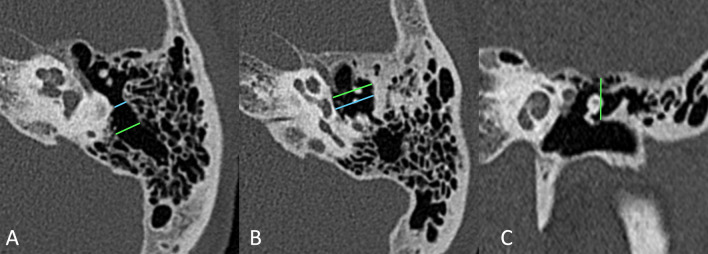
References for measurements of the attico-antral region. Aditus and antrum widths (**A**); anterior and posterior epitympanic widths (**B**); epitympanic height (**C**)

CT volumes have been analyzed using OsiriX MD software [16], and the following parameters were taken into consideration and measured on multiplanar reformats:

1. Mastoid pneumatization, evaluated from reconstructed images on an axial plane (parallel to the lateral semicircular canal where the incudo-malleolar joint and the sigmoid sinus are visible), using the classification proposed by Han et al. [17] in four grades of pneumatization, taking the sigmoid sinus as a reference point (Fig. 1).
2. Prominence of the cog, evaluating its extension in relation to the cochleariform process, starting from the tegmen, on an axial scan; we considered the cog as prominent when its length extended beyond the cochleariform process, and we excluded from the analysis the cases in which, in group A, it was eroded by cholesteatomatous pathology (Fig. 2).
3. Patency of the Eustachian tube, evaluated based on the presence or absence of dense material/mucosal thickening in the protympanic region (Fig. 3).
4. Size of the antrum, measured again on the axial plane, at the point of its maximum width, orthogonal to the major axis (Fig. 4A).
5. Width of the aditus, measured on the same axial plane (Fig. 4A).
6. Width of the epitympanum, measured on an axial plane parallel to the lateral semicircular canal, at two points: one anterior to the neck of the malleus and one posterior at the level of the body of the incus (Fig. 4B).
7. Height of the epitympanum, measured on a coronal plane perpendicular to the one passing through the lateral semicircular canal, from the tegmen to the level of the tympanic annulus in a section passing through the lateral process of the malleus (Fig. 4C).

All measurements were performed by a third-year radiology resident and were reviewed and approved by a radiologist with 6 years of experience in head and neck radiology.

The statistical analysis was performed using IBM SPSS Statistics (IBM Corp. Released 2021. IBM SPSS Statistics for Windows, Version 28.0. Armonk, NY: IBM Corp). Prevalence studies were conducted for discrete variables (degree of mastoid pneumatization, cog prominence, patency of the Eustachian tube), and correlation and group comparison studies were conducted for continuous variables (antrum width, aditus and epitympanic width, epitympanic height).

## Results

Sixty patients have been included in this study, thirty of whom belonged to the group of patients with cholesteatoma and the remaining part to the group of patients without organic otological pathology. The first group included 19 males and 11 females, aged between 9 and 68 years (mean age 35.7 years), while in the second group 16 males and 14 females, aged 12–77 years (mean age 45.1 years), were selected.

**Fig. 5:**
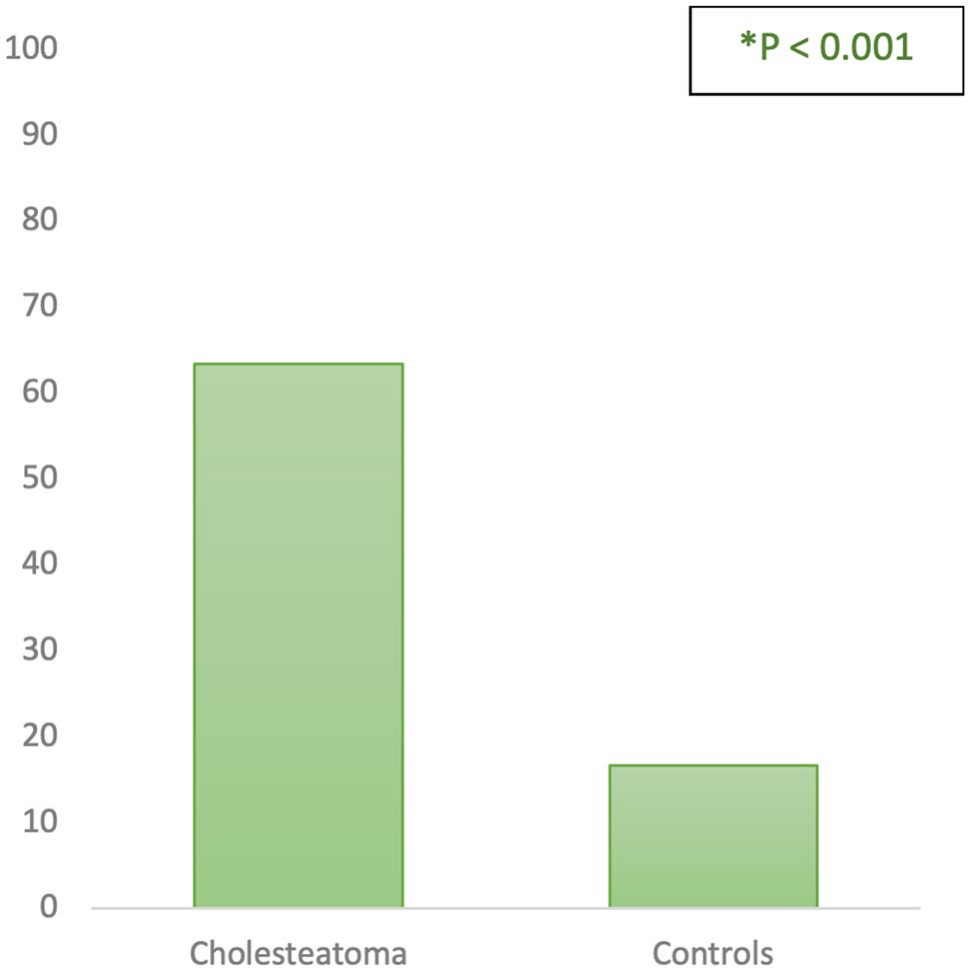
Prevalence of poor mastoid pneumatization

The prevalence of a low degree of mastoid pneumatization among patients with cholesteatoma was found to be present in 19/30 patients (63%), while among subjects in the second group, a low degree of pneumatization was found in only 5/30 cases (17%), with a statistically significant difference between the two groups (*χ*2: 13.6, *p* < 0.001, Fig. 5).

**Fig. 6:**
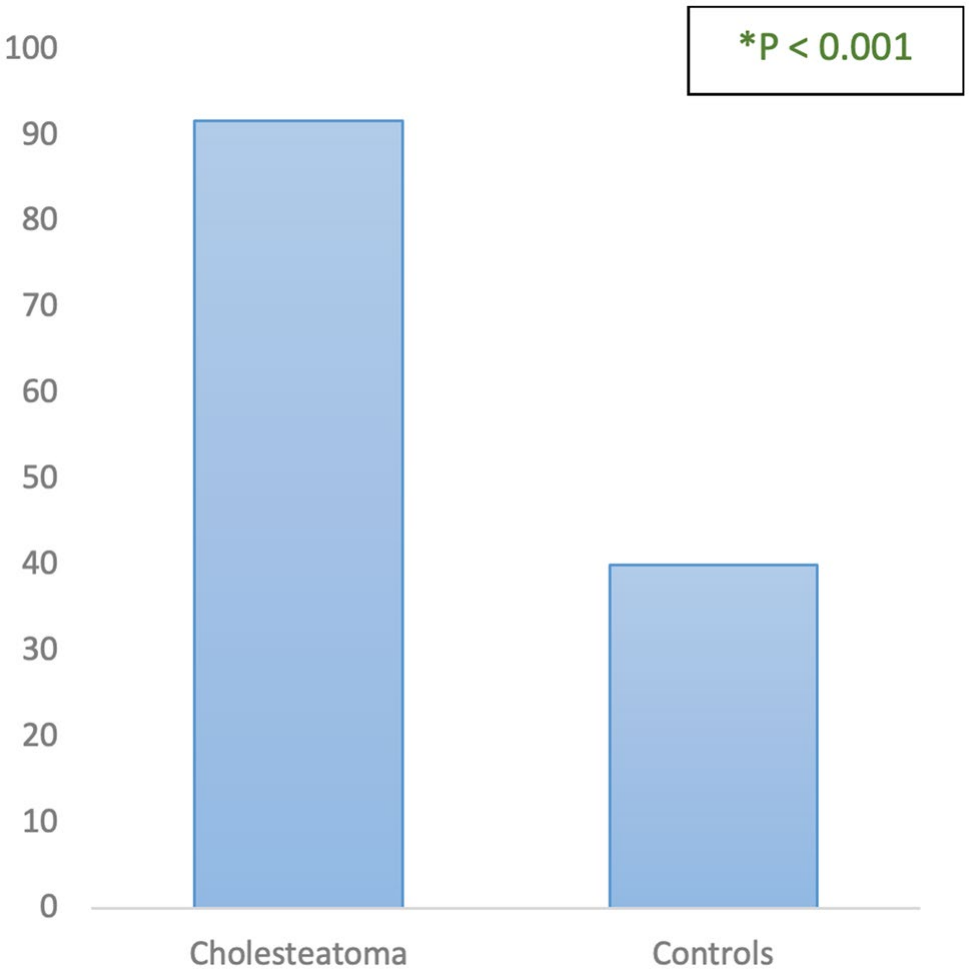
Prevalence of cog prominence

Subsequently, from the group of patients with cholesteatoma, 6 patients were excluded because it was not possible to evaluate the prominence of the cog due to resorption of this bony structure caused by the epitympanic pathology, making it impossible to measure. Comparing therefore the remaining 24 patients affected by cholesteatoma with the 30 controls, it was seen that these two groups differed statistically significantly for the prevalence of cog prominence. In particular, the prevalence of cog prominence among patients with cholesteatoma was 92% (22/24) while that among the control group was 40% (12/30), a statistically significant difference (*χ*2: 15.26, *p* < 0.001, Fig. 6).

The prevalence of tubal obstruction was then studied. It was found that there was no statistically significant difference in tubal obstruction between the two groups. In particular, the prevalence of obstructed tube among subjects with cholesteatoma was 23%, while among subjects in the control group it was only 0.06%, with a non-significant statistical difference (*χ*2: 3.268, *p* = 0.07).

The following results were obtained from the comparison between the two groups for the parameters listed below (Table 1) using the Mann–Whitney test:

- Antrum width (5.5 ± 1.6 mm vs. 5.8 ± 1.6 mm) with a *p* value of 0.329.
- Aditus width (2.6 ± 0.7 mm vs. 2.2 ± 0.5 mm) with a *p* value of 0.035.
- Anterior epitympanic width (4.1 ± 1 mm vs. 6.3 ± 0.8 mm) with a *p* value < 0.001.
- Posterior epitympanic width (5.9 ± 1.21 mm vs. 5.5 ± 0.5 mm) with a *p* value of 0.015.
- Epitympanic height (5.6 ± 1.3 mm vs. 8.2 ± 1.1 mm) with a *p* value < 0.001.

**Table 1:** Measurements of the epitympanic-antral region (dimensions in mm)

	Patients	Healthy subjects	*p* value
Mastoid antrum width	5.5 ± 1.6	5.85 ± 1.6	0.329
Aditus ad antrum width	2.6 ± 0.7	2.2 ± 0.5	0.035
Anterior epitympanic width	4.1 ± 1.0	6.3 ± 0.8	< 0.001
Posterior epitympanic width	5.9 ± 1.2	5.5 ± 0.5	0.015
Epitympanic height	5.6 ± 1.3	8.2 ± 1.1	< 0.001

All the continuous variables measured were found to have statistical significance (*p* < 0.05) in the comparison between groups except for the width of the antrum.

**Fig. 7:**
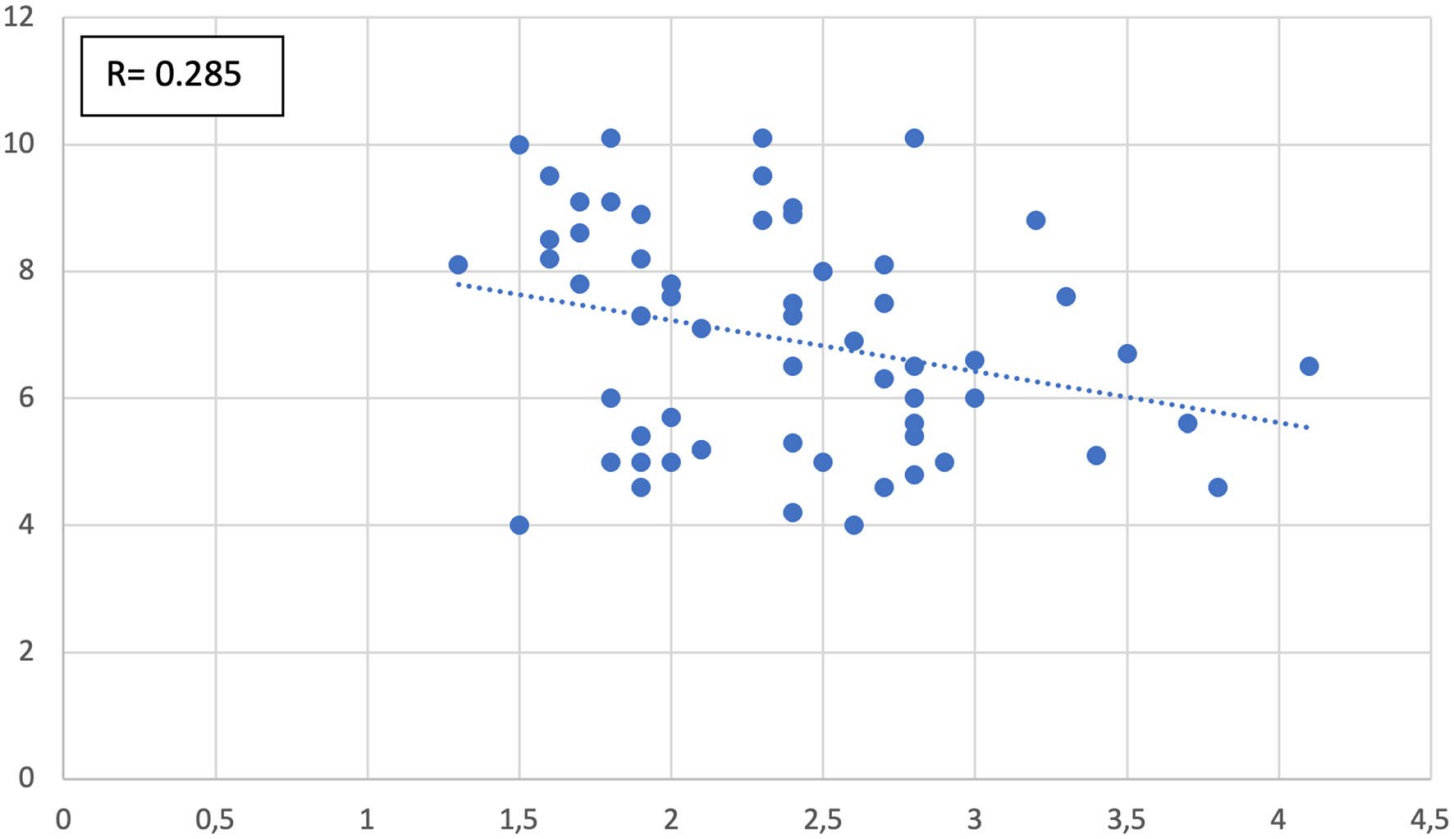
Correlation between antrum width and epitympanum height

**Fig. 8:**
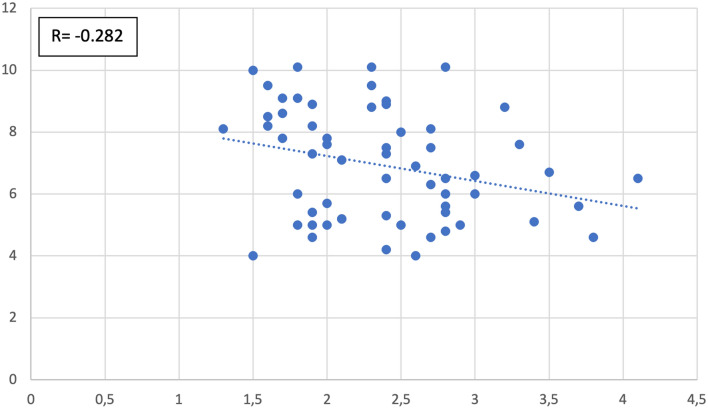
Correlation between aditus width and epitympanum height

**Fig. 9:**
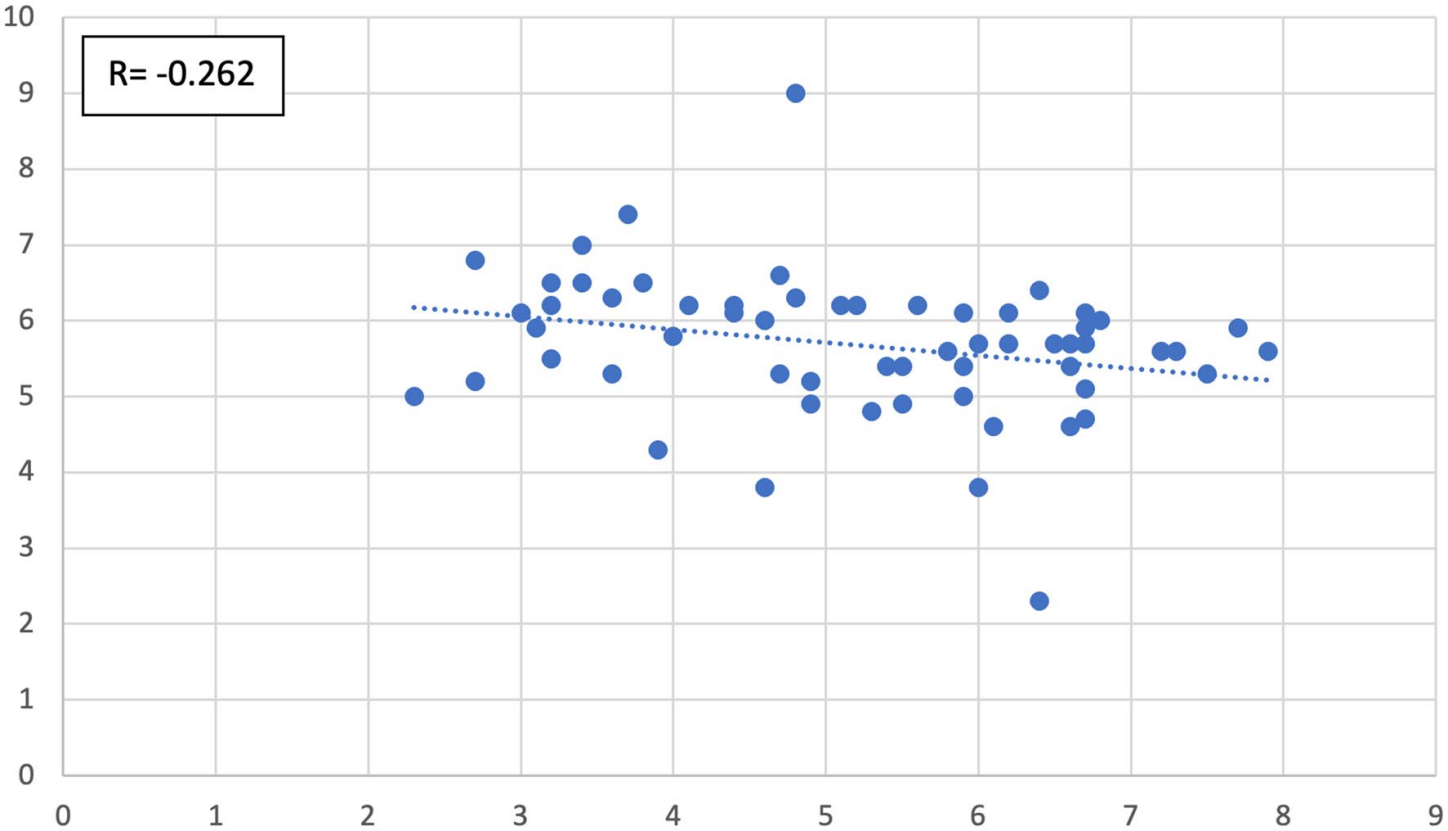
Correlation between width of the anterior epitympanum and posterior epitympanum

**Fig. 10:**
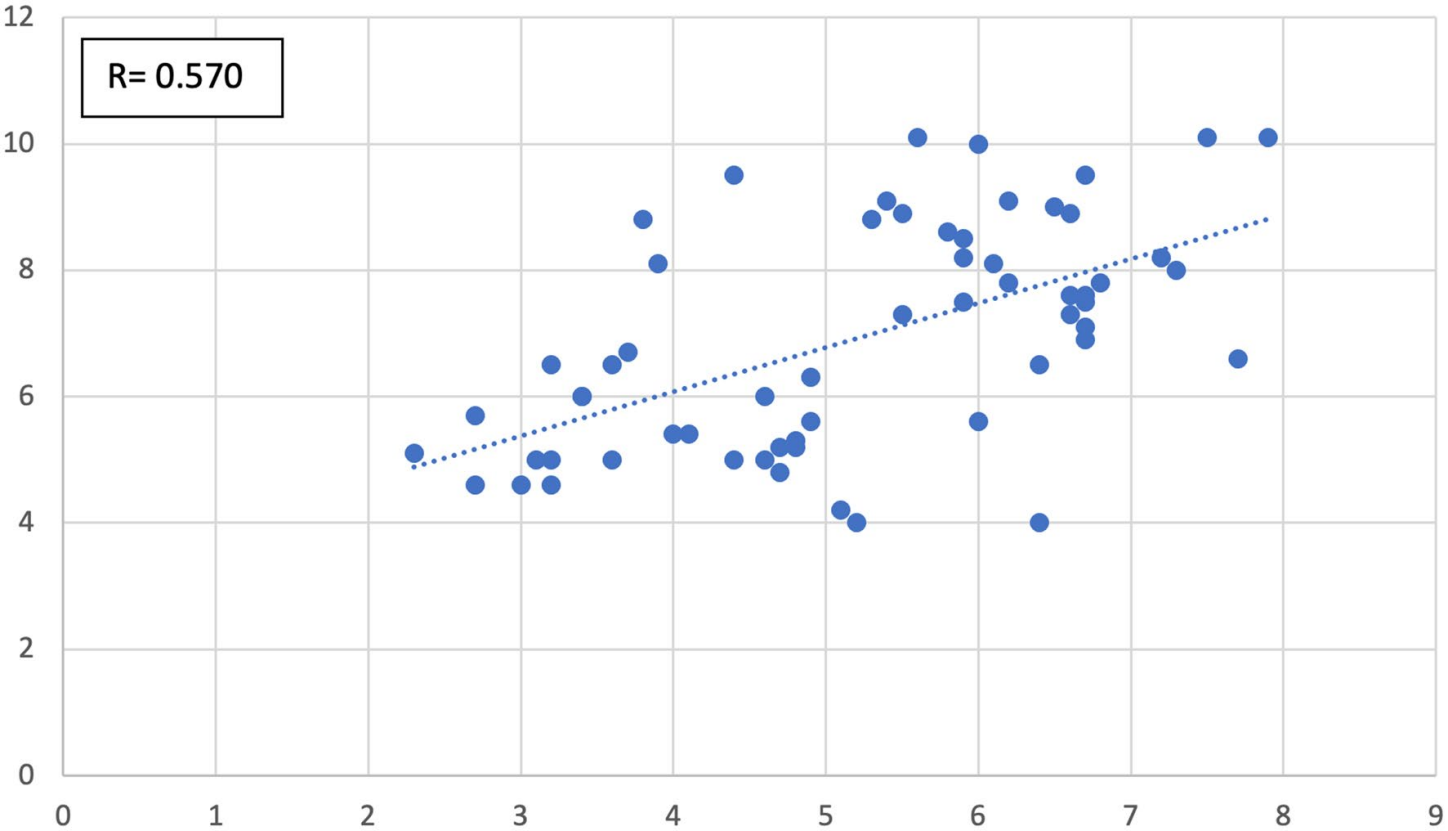
Correlation between anterior epitympanum width and epitympanum height

We also studied, in the entire cohort (cases and controls alike), the type and degree of correlation possibly present among the measured parameters. The continuous variables correlated, significantly, as follows:

- Antrum width positively correlated with epitympanum height (*R* = 0.285 with *p* value = 0.027–Fig. 7).
- Aditus width negatively correlated with epitympanum height (*R* = − 0.282 with *p* value = 0.029–Fig. 8).
- Anterior epitympanum width negatively correlated with posterior epitympanum width (*R* = − 0.262 with *p* value = 0.043–Fig. 9).
- Anterior epitympanum width positively correlated with epitympanum height (*R* = 0.570 with *p* value < 0.001–Fig. 10).

## Discussion

“A gas pocket”, a biological gas pocket: this is how Sadé defined the middle ear, as it contains a mixture of gases similar to those found in the atmosphere [1]. Fink et al. [6] compared the middle ear to a small lung, where the tube is functionally organized like a mini-trachea, and the mastoid pneumatic system is compared to alveoli. In the context of this physiological model, the pneumatic system represents the district responsible for the transmucosal diffusion of gases dissolved in the blood perfusing the mastoid mucosa, so a well-pneumatized mastoid, in which the number of cells is high, guarantees an adequate exchange surface and contributes to proper ventilation of the tympanic cavity [18]. On the contrary, it is a common clinical observation that in patients with cholesteatoma, and generally with chronic otitis media, there is a concomitant mastoid hypopneumatization. However, it is not clear whether this hypopneumatization is the cause or the effect of chronic otitis media [19]. Some authors argue that recurring inflammations in childhood and/or tubal occlusion [20] are the cause of reduced mastoid pneumatization, while other studies conducted on animal models have shown that mastoid pneumatization continues even in the presence of tubal ventilation blockage in early age [21] and that it is never complete before the age of 8–9 years [22]. Other studies still believe that the extent of mastoid pneumatization has a genetic basis. Evidence of this is the identical degrees of mastoid pneumatization in monozygotic twins and the hyperpneumatization of the mastoid that occurs in otopathies of genetic origin, such as otosclerosis itself [23]. According to the literature, our study also revealed that a reduced degree of mastoid pneumatization correlates positively with the presence of cholesteatoma. In fact, our study found that a poorly pneumatized mastoid is present in almost two-thirds of the population with cholesteatoma, while it is found in only just over 16% of healthy subjects, with a statistically significant difference between the two groups. However, it is worth noting that it is not possible to speculate on the cause or effect role of reduced mastoid pneumatization on the development of cholesteatoma, as it was not possible to analyze data longitudinally.

In addition to the mastoid, the Eustachian tube also plays a fundamental role in ensuring proper ventilation of the middle ear. The function of the Eustachian tube is to compensate for the pressure gap that would occur if aeration of the middle ear were solely dependent on gas exchange through the mucous membrane. The latter is only capable of ensuring an intratympanic pressure of 724 mmHg, while the Eustachian tube, thanks to the inhalation of air from the nasopharynx (1–2 ml/24 h), enriches the intratympanic pressure with the remaining 36 mmHg, in order to balance atmospheric pressure and avoid retractions of the tympanic membrane [24]. In pathological conditions, the activity of the Eustachian tube may be impaired due to dysfunctional phenomena or obstructions at the nasopharyngeal level (adenoid hypertrophy, neoplasms), intrinsic to the Eustachian tube itself or at the level of the tympanic ostium. In our study, for methodological reasons (analysis of CT data), we were unable to evaluate possible dysfunctional phenomena, nor did we find any nasopharyngeal obstructive causes. In accordance with literature data, we considered the radiological measurement of the bony portion of the Eustachian tube to be unreliable, so we only evaluated the presence of an obstruction of the tubal orifice at the level of the protympanum [25]. As expected, a higher number of cases of tubal obstruction were found in the population affected by cholesteatoma compared to the control population, but this difference was not statistically significant. These data demonstrate the secondary role of the Eustachian tube compared to mastoid pneumatization in determining a chronic condition such as cholesteatoma, as the Eustachian tube is more involved in the pathogenesis of acute pathologies. Greater importance for the establishment of cholesteatomatous pathology is instead given to the obstacles to endotympanic ventilation currents: a prominent cog, an obstructed tympanic isthmus, the presence of tensor folds, as well as reduced dimensions of both the anterior and posterior epitympanum, aditus, and antrum, which may represent predisposing conditions for the development of cholesteatoma.

The main limitation of our study was not having endoscopic data available in the two groups regarding the possible presence of mucosal folds, but all the other bone variables considered were statistically significant in influencing the genesis of cholesteatoma, except for the size of the antrum, probably because the latter represents the mastoid cell characterized by the most constant dimensions in the general population. In the population of patients with cholesteatoma, we observe a statistically significant reduction in the values of height and width of the epitympanum and also of the aditus ad antrum. Furthermore, in a correlation analysis, the two parameters (height and width of the anterior epitympanum) were found to be the most significantly correlated, meaning that as the height of the epitympanum decreases, the width of the epitympanum also decreases proportionally, resulting in a final effect of a reduction in epitympanic volume, which clinically translates into less effective ventilation. Another limitation of our study is that the control group may not be fully representative of the general population, as it consists of patients who underwent temporal bone CT for others and various reasons.

## Conclusion

In line with the current literature, this study confirms that there is a significant prevalence of “dysfunctional mastoid” (i.e., poorly pneumatized) in patients with cholesteatoma compared to controls, in whom good levels of pneumatization are usually encountered. Furthermore, in the middle ear affected by cholesteatoma, a significant reduction in all the epitympanic measures could be a good radiological correlate of the poor ventilatory capabilities of the epitympanum in these patients, while the contribution of tubal obstruction has been found to be less significant. A complete understanding of the impact of the anatomical and physiological mechanisms of middle ear ventilation is a prerequisite for the proper management of the pathology and better control of its recurrences. The future goal should be to integrate the radiological data considered in this study with endoscopic findings with a prospective study design and longitudinal assessment.
